# CYLD in health and disease

**DOI:** 10.1242/dmm.050093

**Published:** 2023-06-30

**Authors:** José L. Marín-Rubio, Ishier Raote, Joseph Inns, Carol Dobson-Stone, Neil Rajan

**Affiliations:** ^1^ Translational and Clinical Research Institute, Newcastle University, Newcastle upon Tyne NE1 7RU, UK; ^2^ Université de Paris, CNRS, Institut Jacques Monod, Paris 75016, France; ^3^ Brain and Mind Centre and School of Medical Sciences, University of Sydney, Sydney, NSW 2050, Australia; ^4^ Department of Dermatology, Royal Victoria Infirmary, Newcastle upon Tyne NE1 4LP, UK

**Keywords:** CYLD, Cylindroma, Frontotemporal dementia, Genetics, Skin tumours, Ubiquitination

## Abstract

CYLD lysine 63 deubiquitinase (CYLD) is a ubiquitin hydrolase with important roles in immunity and cancer. Complete *CYLD* ablation, truncation and expression of alternate isoforms, including short CYLD, drive distinct phenotypes and offer insights into CYLD function in inflammation, cell death, cell cycle progression and cell transformation. Research in diverse model systems has shown that these are mediated via CYLD regulation of cellular pathways including the NF-κB, Wnt and TGF-β pathways. Recent biochemical advances and models have offered new insights into the regulation and function of CYLD. In addition, recent discoveries of gain-of-function germline pathogenic *CYLD* variants in patients with a neurodegenerative phenotype contrast with the more widely known loss-of-function mutations seen in patients with CYLD cutaneous syndrome and with sporadic cancers. Here, we provide a current review of mechanistic insights into CYLD function gained from *CYLD* animal models, as well as an update on the role of CYLD in human disease.

## Teaser

**Summary:** Advances in the study of the cylindromatosis gene (*CYLD*), utilising diverse models, provide new insights into cellular homeostasis and human diseases.

## Introduction

CYLD lysine 63 deubiquitinase (CYLD) is a ubiquitin hydrolase enzyme that removes ubiquitin molecules from other proteins (Box 1), and is critical in regulating various signalling pathways that are involved in inflammation, immunity, cell survival and cancer. *CYLD* was isolated more than 20 years ago (Bignell et al., 2000) and was initially associated with the rare disease familial cylindromatosis [Online Mendelian Inheritance in Man (OMIM) 132700] (Biggs et al., 1995). Further work implicated *CYLD* pathogenic variants in a broader spectrum of inherited skin tumour syndromes, now collectively named CYLD cutaneous syndrome (CCS) (Dubois and Rajan, 2020).

**Box 1. Ubiquitination**

Ubiquitination is an important and abundant post-translational modification, particularly in eukaryotic cells. Although protein ubiquitination is traditionally linked to proteasomal degradation by the ubiquitin–proteasome system, many proteins are regulated by non-degradative ubiquitin and ubiquitin-like signals, which can dictate protein−protein interactions, localisation and enzymatic activity, thereby influencing transcription, cell cycle progression and other functions critical for homeostasis (Herrmann et al., 2007; Swatek and Komander, 2016). Ubiquitination is a dynamic and reversible process catalysed by a cascade of three ubiquitin-modifying enzymes that covalently attach ubiquitin to cellular proteins. This can be mediated by a single or several ubiquitin molecules (monoubiquitination or polyubiquitination), with the latter occurring in linear or branched architectures (Akutsu et al., 2016; Mevissen and Komander, 2017). Polyubiquitin chains contain several ubiquitin molecules, covalently linked by their N-terminal methionine residue (M1) into a linear chain or by one of their seven lysine residues – K6, K11, K27, K29, K33, K48 and K63 – into an array of polyubiquitin conformations (Akutsu et al., 2016; Mevissen and Komander, 2017). Ubiquitination is reversed by ubiquitin proteases or deubiquitinating enzymes, such as CYLD, that remove ubiquitin, polyubiquitin chain types or ubiquitin-like proteins, including SUMO, NEDD8 and ISG15 (Geurink et al., 2019; Kerscher et al., 2006; Zheng and Shabek, 2017).

Dysregulation or dysfunction of CYLD has indeed been implicated in a number of human diseases, including, but not limited to, cancer, infectious disease and multiple neurodegenerative disorders (Dai et al., 2022; Fraile et al., 2012; Kowalski and Juo, 2012). For these reasons, CYLD is currently a target for therapeutic development. Although researchers have uncovered several aspects of CYLD structure and function in the intervening years, particularly through the use of diverse model systems, our understanding of its biology and role in disease remains incomplete.

In this Review, we summarise the broad array of cellular functions of CYLD, the effects of pathogenic variants on said functions and how these effects drive human disease. We also discuss emerging questions in this field, particularly those relating to clinical translation.

## CYLD expression and protein structure

**Fig. 1.**
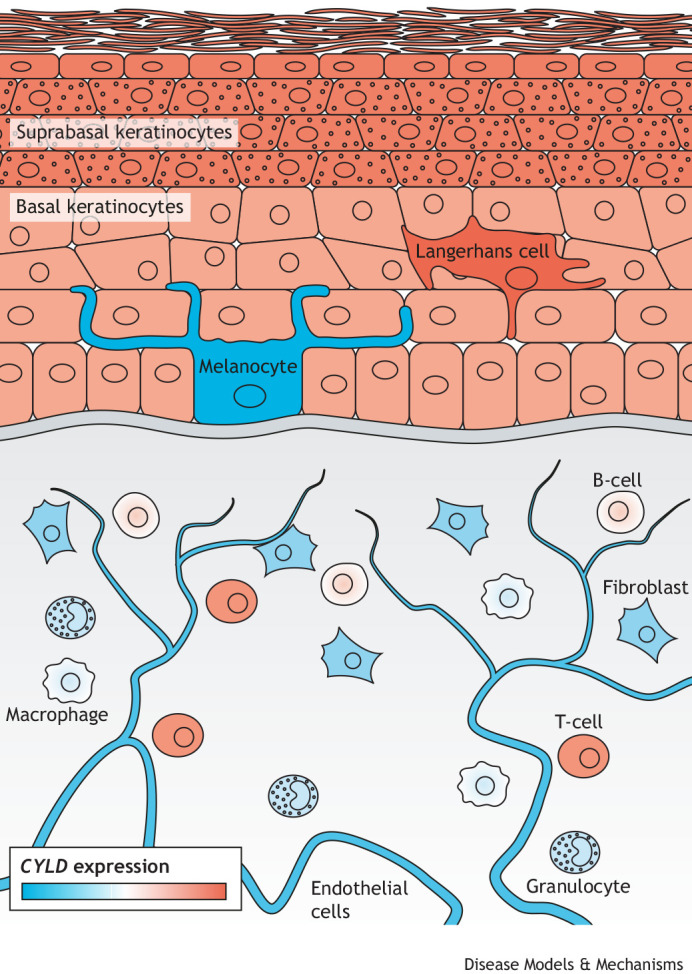
***CYLD* expression in normal human skin.** *CYLD* is expressed in diverse human skin cells. Here, we show expression levels in individual skin cell types as measured by single-cell RNA sequencing from the Human Protein Atlas database (Uhlen et al., 2010) (https://www.proteinatlas.org/ENSG00000083799-CYLD/single+cell+type). The blue-to-red gradient represents lowest-to-highest transcripts per million (TPM). The highest level of *CYLD* expression was measured in suprabasal keratinocytes, Langerhans cells and T-cells (red); the lowest expression of *CYLD* was found in melanocytes (blue).

CYLD is located on chromosome 16q12.1 in humans and consists of 20 exons that generate 15 transcript variants, but only three protein isoforms. Full-length CYLD is composed of 20 exons, which encode a protein of 956 amino acids. A shorter isoform of 953 amino acids is encoded by splice variants missing the very short exon 7 (9 bp) (Bignell et al., 2000). In mice, a third 770 amino-acid isoform, named ‘short CYLD’ (sCYLD), results from skipping of exons 7 and 8 (Hahn et al., 2018; Tang et al., 2019). *CYLD* is highly expressed in suprabasal and basal keratinocytes, Langerhans cells, oligodendrocytes, blood and immune cells (Fig. 1), highlighting the importance of this gene in several skin cell types and their functions. *CYLD* is also expressed in foetal brain, testis and skeletal muscle, and at a lower level in adult brain, leukocytes, liver, heart, kidney, spleen, ovary and lung (Bignell et al., 2000).

*CYLD* expression and function are regulated through diverse mechanisms. *CYLD* expression is reduced by methylation of CpG islands in its promoter or by microRNA (miRNA)-mediated silencing. For example, the *CYLD* promoter is methylated in gastric cancer (Ghadami et al., 2019). Moreover, hypermethylation of the *CYLD* promoter may be influenced by infectious agents such as *Helicobacter pylori*, Epstein-Barr virus and cytomegalovirus (Ghadami et al., 2019). According to miRTarBase, there are at least five miRNAs that might target human *CYLD*: miR-181b, miR-182, miR-130b, miR-362 and miR-500a. Another mechanism that reduces *CYLD* expression is BRAF-mediated ERK (MAPK) activation via the transcription factor SNAIL1 (SNAI1) in melanoma (Massoumi et al., 2009). Conversely, serum promotes *CYLD* expression through the recruitment of serum response factors to serum response element sites located in the *CYLD* promoter in a p38 MAPK activation-dependent manner (Liang et al., 2011).

**Fig. 2.**
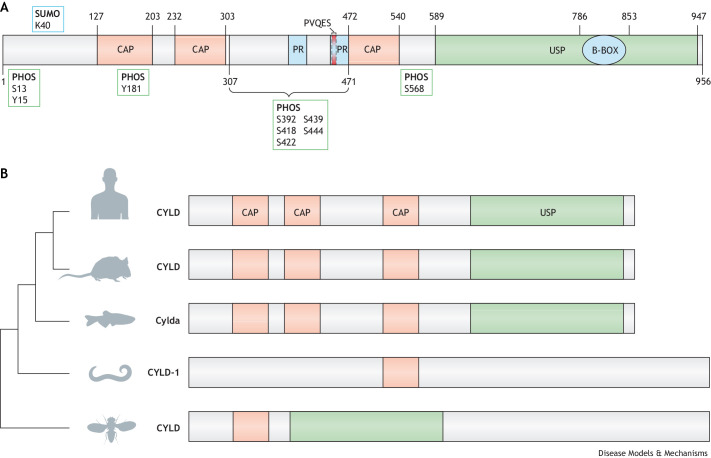
**CYLD structure, conservation and genetic models.** (A) The N-terminal portion of CYLD has three glycine-rich cytoskeleton-associated protein (CAP-Gly) domains. The first, CAP-Gly1 [amino acids (aa) 127-203], is necessary for CYLD association with microtubules (Gao et al., 2008; Yang et al., 2015), while the CAP-Gly3 domain (aa 472-540) binds the nuclear factor kappa-B (NF-κB) essential modifier (NEMO/IKK-γ) that allows modulation of inflammatory NF-κB signalling (Saito et al., 2004). CYLD also contains a TRAF-binding motif (PVQES) (aa 453-457) and two proline-rich motifs (PR) (aa 388-413, 446-471). The C-terminal portion of CYLD contains a zinc-finger like B-box domain (B-BOX; aa 786-853) within the ubiquitin-specific protease (USP) domain. PHOS, phosphorylation sites; SUMO, SUMOylation site. (B) Phylogenetic analysis of CYLD protein and protein domain architecture in human, *Mus musculus*, *Dario rerio*, *Caenorhabditis elegans* and *Drosophila melanogaster*. CAP, cytoskeleton-associated protein.

CYLD is one of ∼100 different human deubiquitinating enzymes (DUBs; Box 1). In particular, CYLD belongs to the family of ubiquitin-specific protease (USP) cysteine proteases (Mevissen and Komander, 2017) and cleaves K63- and M1-linked polyubiquitin chains (Ang et al., 2021; Komander et al., 2009; Priem et al., 2019). In addition, some activity has been demonstrated against K11- and K48-linked polyubiquitin chains (Komander et al., 2008; Sato et al., 2015). The CYLD protein is made up of three glycine-rich cytoskeleton-associated protein (CAP-Gly) domains, two proline-rich motifs, a phosphorylation region and the USP catalytic domain. A zinc-finger-like B-box domain also exists within the USP domain, which is important for the subcellular localisation of CYLD (Bignell et al., 2000; Komander et al., 2008) (Fig. 2A). The C-terminal portion of CYLD (amino acids 470-957) is known to bind BCL-3 and contains the catalytic domain (Massoumi et al., 2006). Patients with CCS typically carry truncating pathogenic variants that are catalytically inactive, for example, only encoding amino acids 1-936 (Saito et al., 2004; Trompouki et al., 2003). The DUB activity of CYLD can also be regulated through post-translational modifications (Fig. 2A). Phosphorylation at S418 weakens the catalytic efficiency of CYLD, which impairs the degradation of its substrates TRAF2 and TRAF6 and, consequently, promotes NF-κB signalling. This phosphorylation is carried out by members of the IκB kinase (IKK; IKBK) family, including NEMO (IKBKG)/IKKα (CHUK), IKKβ (IKBKB), TBK1 and the non-canonical IKK kinase IKKε (IKBKE) (Hutti et al., 2009; Lork et al., 2018; Reiley et al., 2005). However, phosphorylation at both S418 and S568 by IKKβ after TNF stimulation activates CYLD K63-linked ubiquitin DUB activity (Elliott et al., 2021). TNF stimulation also induces the phosphorylation of several other serines (S392, S418, S422, S439, S444) within the phospho-rich patch of CYLD (Elliott et al., 2021). Similarly, SUMOylation of CYLD at K40 can reduce the efficiency of CYLD-mediated deubiquitination of TRAF2 and TRAF6 (Kobayashi et al., 2015). Oxidation can also inhibit the catalytic activity of CYLD (Wang and Wang, 2021), meaning that disruption of CYLD through oxidative stress could enhance inflammation. Moreover, prolonged hypoxia in human papillomavirus (HPV)-positive cancer cells mediates the stimulation of the HPV-encoded E6 protein, which induces polyubiquitination and proteasomal degradation of CYLD (An et al., 2008). In addition, non-DUB functions of CYLD have been reported. In mitosis, for example, CYLD interacts with histone deacetylases at the midbody to regulate the rate of cytokinesis in a DUB-independent manner (Wickström et al., 2010). These findings suggest that CYLD is embedded in a complex regulatory network that underlies its diverse cellular functions and may help explain its role in a number of diseases.

The evolutionary conservation of this protein across species (Fig. 2B) is an essential aspect that allows researchers to study CYLD in different model systems. *CYLD* is an evolutionarily conserved gene, and orthologues have been described in *Drosophila melanogaster* and *Caenorhabditis elegans* (Hadweh et al., 2018). All orthologues have highly conserved USP domains (Hu et al., 2002; Young et al., 2019; Komander et al., 2008). Similarly, there are several specific phosphorylation sites in common in vertebrates and mammals (Elliott et al., 2016; Kovalenko et al., 2003; Reiley et al., 2005), indicating conserved regulation of this protein. Cross-species comparison can offer mechanistic understanding of changes in protein function, particularly those associated with mutations in conserved domains that could be teased apart in model organisms (Box 2). Despite the conservation of CYLD, model organisms may not always recapitulate equivalent regulation. An example of the importance of model selection is illustrated by the pathogenic variant M719V in CYLD, which is associated with frontotemporal dementia (FTD) in humans (Dobson-Stone et al., 2020). This pathogenic variant increases K63 deubiquitinase activity and therefore increases the inhibition of NF-κB (Dobson-Stone et al., 2020). Unusually, the corresponding amino acid in *Drosophila* dCYLD (CYLD) is already a valine, rendering *Drosophila* unsuitable for the study of this particular disease-causing CYLD variant. Therefore, specific functions and/or CYLD variants can only be studied in mammalian models or in humanised transgenic non-mammalian models. Researchers have generated a range of mouse models to understand CYLD variants relevant to human pathology (Table 1). In addition, the large number of targets of CYLD activity and their diverse functional proteostatic consequences complicate our ability to appreciate conserved functions in different models. For this reason, model contextualisation is important in the study and interpretation of the diversity of CYLD functions (Fig. 2C).

**Box 2. Modelling CYLD**

CYLD function was initially studied in cell lines (Brummelkamp et al., 2003; Komander et al., 2008; Stegmeier et al., 2007; Trompouki et al., 2003; Kovalenko et al., 2003; Brummelkamp et al., 2003; Komander et al., 2008; Stegmeier et al., 2007; Trompouki et al., 2003) and subsequently modelled in mice (Massoumi et al., 2006), *Drosophila* (Chen et al., 2014; Xue et al., 2007), zebrafish (Li et al., 2021a), rainbow trout (Jang et al., 2020), worms (Hadweh et al., 2018) and, most recently, crabs (Zhang et al., 2022).

Animal models of CYLD function can play a critical role in understanding how CYLD functions and can reveal interactions with other cellular modules. In mice, a range of modelling strategies has been employed (Table 1). Initial strategies that targeted the initiation codon led to total *Cyld* knockout, with viable *Cyld*^−/−^ mice demonstrating an increased predisposition to tumour formation in chemical carcinogenesis models (Massoumi et al., 2006). Subsequently, truncating mutations that mimicked the human CYLD cutaneous syndrome (CCS) patient genotypes were modelled, and this resulted in perinatal lethality of homozygous mice that ubiquitously expressed the transgene, owing to abnormalities of lung maturation (Trompouki et al., 2009).

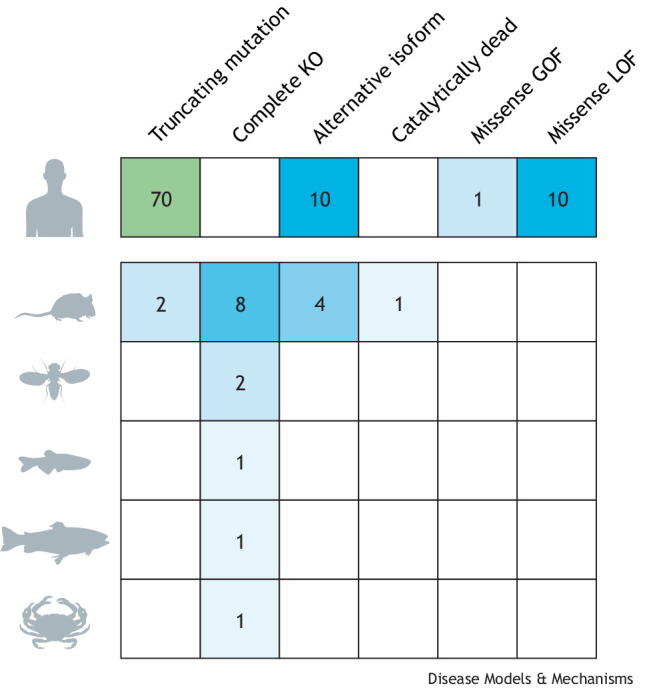

**Number of animal models with different genetic strategies for studying CYLD function, with human CCS pathogenic variants shown for comparison.** Blank squares represent a lack of models. GOF, gain of function; KO, knockout; LOF, loss of function.

The naturally occurring murine alternatively spliced short isoform termed *sCyld* has also been modelled by an exon 7-targeting strategy that removes the TRAF2- and NEMO-binding sites (Hövelmeyer et al., 2007). Cre/loxP recombination strategies have subsequently offered specific insights into the roles of CYLD in individual cell types, including T-cells (Reuter et al., 2016), dendritic cells (Srokowski et al., 2009) and hepatocytes (Urbanik et al., 2012).

**Table 1.**
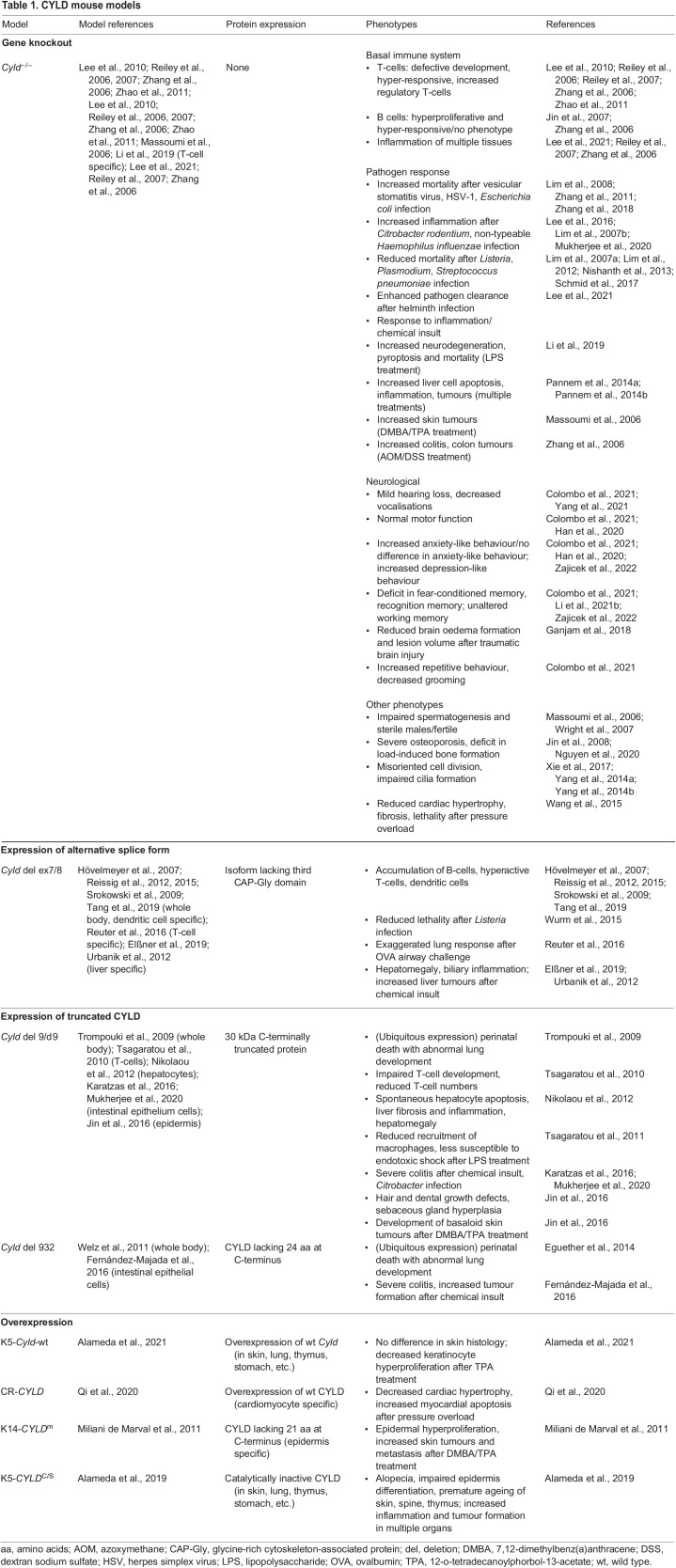
CYLD mouse models

## Role of CYLD as a tumour suppressor and a regulator of cellular processes

**Fig. 3.**
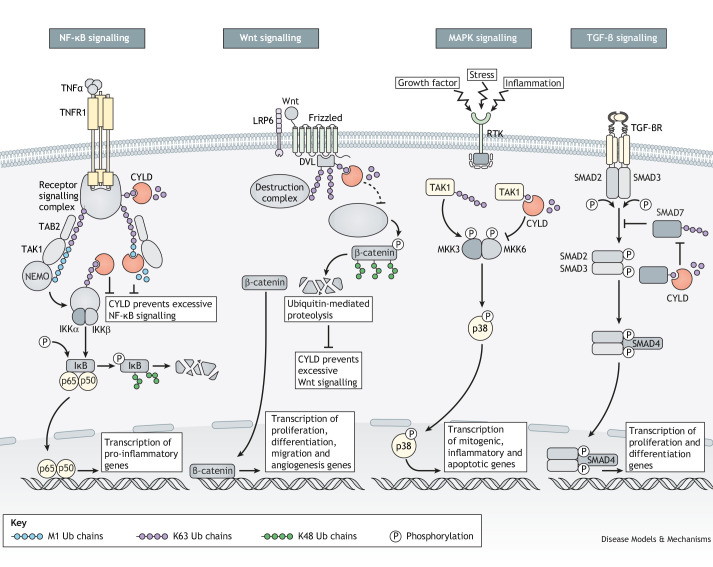
**CYLD regulates cell signalling pathways through deubiquitination of effector proteins.** CYLD regulates a range of signalling pathways through its deubiquitinase activity, targeting K63- and M1-linked polyubiquitin chains of effector proteins. CYLD represses NF-κB signalling by deubiquitinating receptor signalling complex proteins such as those associated with the TNFα receptor, as well as its downstream effectors such as TAB2. In Wnt signalling, CYLD deubiquitinates dishevelled (DVL) to prevent the sequestering of β-catenin, thereby reducing Wnt signal transduction. CYLD also affects regulation of MAPK signalling by targeting K63 polyubiquitination of TAK1 after receptor tyrosine kinase (RTK) activation. Thereby, CYLD impedes p38-mediated gene transcription. Finally, CYLD promotes TGF-β signalling through deubiquitination of SMAD7, thereby preventing SMAD7-mediated inhibition of SMAD2/3 phosphorylation (P). K48 Ub, K48-linked ubiquitin; K63 Ub, K63-linked ubiquitin; M1 Ub, M1-linked ubiquitin.

The requirement of ubiquitin chains for NF-κB activation was discovered nearly 30 years ago (Chen et al., 1995; Palombella et al., 1994; Traenckner et al., 1994). In vertebrates, this is mediated by the linear ubiquitin chain assembly complex (LUBAC), which is the only known E3 ligase complex that catalyses the addition of M1-ubiquitin chains to substrates such as NF-κB. LUBAC is composed of three core subunits: HOIL-1 (RBCK1), HOIP (RNF31) and SHARPIN (Gerlach et al., 2011; Kirisako et al., 2006). In addition to modifying branched ubiquitin, CYLD and OTULIN are the two DUBs known to regulate LUBAC and, subsequently, inflammation and immune signalling (Sato et al., 2015). The recruitment of CYLD to LUBAC occurs via an indirect interaction with HOIP mediated by SPATA2, which has also been shown to activate CYLD DUB activity (Elliott et al., 2016; Sato et al., 2015). Therefore, CYLD is an important negative regulator of NF-κB via its deubiquitination targets TRAF2, TRAF6, TAK1 (NR2C2) and NEMO (Brummelkamp et al., 2003; Kovalenko et al., 2003; Reiley et al., 2007; Trompouki et al., 2003) (Fig. 3). Moreover, CYLD regulates a range of cell survival pathways, including the Wnt/β-catenin, MAPK and TGF-β signalling pathways (Fig. 3). Also, CYLD is a crucial regulator of diverse cellular processes such as inflammation, cell death, cell cycle progression and malignant transformation (Legarda et al., 2016; Moquin et al., 2013; O'Donnell et al., 2011; Priem et al., 2019; Sun, 2010; Wright et al., 2007). These processes are mediated in some contexts by regulating specific signal transduction pathways, in which CYLD interacts with and deubiquitinates proteins important for several cellular functions. Several of these signalling pathways are essential in maintaining cell and tissue homeostasis and can, when dysregulated by CYLD loss, result in malignancy. This underscores CYLD's role as a tumour suppressor regulating two important hallmarks of cancer: cell death and proliferation.

### Role of CYLD in cell death and autophagy

**Fig. 4.**
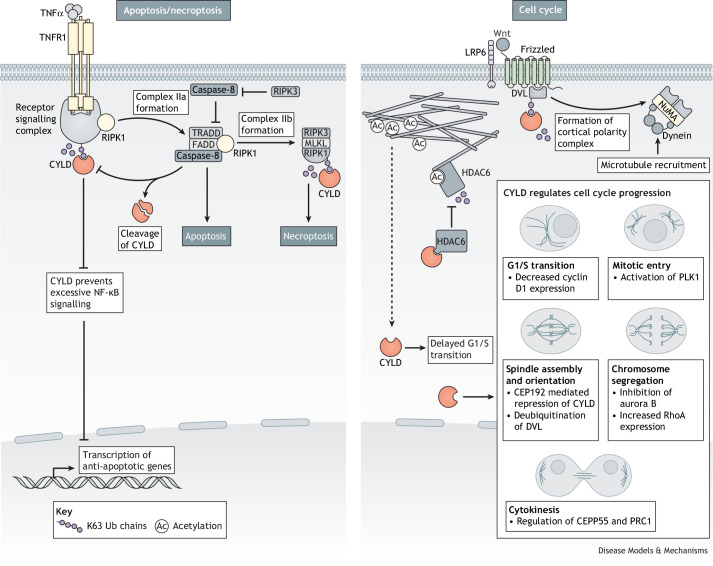
**CYLD regulates integral cellular activities including apoptosis and necrosis as well as the cell cycle.** CYLD regulates critical cell death programs, apoptosis and necroptosis, through K63 deubiquitination of RIPK1. Additionally, CYLD has numerous regulatory roles throughout the cell cycle. These include deubiquitination of target proteins such as DVL and PLK1, as well as direct interactions with cell cycle effectors HDAC6 and CEP192. K63 Ub, K63-linked ubiquitin.

CYLD has been identified as a master switch between apoptosis, the classical cell death pathway, and necroptosis, a form of programmed cell death involving cell membrane permeabilisation and cell lysis (Hitomi et al., 2008; O'Donnell et al., 2011). This interplay has been best characterised in the pro-cell-death TNF signalling pathway and its signal transduction kinase RIPK1. Deubiquitination of RIPK1 by CYLD inhibits NF-κB signalling and promotes the RIPK1-mediated engagement and activation of caspase-8, which triggers apoptosis (Fig. 4). In turn, caspase-8 can cleave and inactivate CYLD (O'Donnell et al., 2011), thus forming a negative feedback loop that enables a balance between pro-survival and pro-apoptotic processes. However, if caspases are inhibited, CYLD deubiquitination of RIPK1 enables its interaction with RIPK3 and MLKL to form the necrosome and trigger necroptosis (Moquin et al., 2013; Vanlangenakker et al., 2011) (Fig. 4). CYLD can also mediate necroptosis independently of TNF receptors via TLR3 and TLR4 (Schworer et al., 2014) or via RIG-1 (Schock et al., 2017), and can mediate necroptosis-like cell death in response to oxidative stress in neuronal cells (Ganjam et al., 2018).

Conversely, CYLD inhibits cell death in certain contexts. For example, in *Drosophila*, dCYLD regulates TNF-induced activation of JNK pathways and cell death through dTRAF2 (Traf6) and the kinase dTAK1 (Tak1). dCYLD deubiquitinates dTRAF2, reducing its degradation and thus maintaining the pro-apoptosis signal (Xue et al., 2007). CYLD also prevents spontaneous apoptosis of periportal hepatocytes by inhibition of TAK1 (Nikolaou et al., 2012). Lastly, CYLD inhibits pyroptosis, a pro-inflammatory form of cell death mediated by caspase-1 in response to lipopolysaccharide stimulation (Li et al., 2019).

Autophagy is fundamental to cellular homeostasis, responsible for clearing misfolded proteins or turning over damaged organelles. CYLD plays roles in controlling the stability and signalling of several proteins, resulting in cell- and condition-dependent effects on autophagy. Specifically, CYLD functions as a DUB for AKT and mTOR, directly controlling and promoting autophagic flux (Colombo et al., 2021; Zajicek et al., 2022; Qi et al., 2020). In particular, autolysosome fusion is impaired in human embryonic kidney cells overexpressing mutant CYLD_M719V_ (Dobson-Stone et al., 2020). CYLD inhibits the formation of autophagosomes by regulating RIPK2-mediated ERK1/2 (MAPK3/1) activity in macrophages (Wex et al., 2015). Autophagic recycling of intracellular aggregates is mediated by protein aggregate sensor receptors including p62, HDAC and NBR1, all of which are regulated by CYLD (Mathis et al., 2015; Mitra et al., 2011; Willis et al., 2010). The CYLD-associated E3 ubiquitin ligase TRAF6 interacts with autophagy regulatory proteins, such as HO1 (HMOX1), HMGB1 and beclin-1, further regulating cellular autophagic responses (Banerjee et al., 2012; Tang et al., 2010; Wirawan et al., 2010).

Therefore, CYLD not only controls inflammation mediated by NF-κB but can also control different types of cell death that can affect inflammation, such as necroptosis and pyroptosis, as well as autophagy.

### Role of CYLD in cell cycle control

CYLD is required for efficient entry into mitosis, in part due to interaction with PLK1 (Stegmeier et al., 2007). Additionally, CYLD activation increases the levels of acetylated α-tubulin by interaction of the N-terminal CAP-Gly domain of CYLD with the catalytic site of HDAC6, inhibiting HDAC6-mediated tubulin deacetylation. In turn, acetylated α-tubulin promotes CYLD translocation to the perinuclear region, where CYLD induces a delay in the G1/S transition of the cell cycle through a BCL-3-mediated pathway. Mitotic spindle formation is regulated by CYLD's direct interaction with CEP192 (Gao et al., 2008; Gomez-Ferreria et al., 2012) (Fig. 4). CYLD controls spindle orientation both via its role as microtubule-associated protein and as a deubiquitinase. Mitotic spindle orientation relative to the cell cortex controls how the cell division plane is oriented, thereby contributing to tissue organisation. CYLD interacts with PLK1 and regulates its activity by deubiquitinating K63-linked ubiquitin chains on PLK1 or its upstream regulators (Stegmeier et al., 2007) (Fig. 4). CYLD also deubiquitinates K63-linked ubiquitin chains on DVL, promoting the DVL–NUMA–dynein/dynactin complex formation at the cell cortex, which is required to generate pulling forces on astral microtubules (Tauriello et al., 2010) (Fig. 4). Concomitantly, CYLD stabilises astral microtubules, defining how microtubules interact with cortical sites. Accordingly, intestinal crypt cells in *Cyld*^−/−^ mice show altered spindle orientation relative to that of wild-type mice (Yang et al., 2014a). In *Drosophila*, dCYLD associates with Hpo, a core component of the *Drosophila* Hippo pathway recognised to control spindle orientation and asymmetric cell division (Keder et al., 2015). The association reduces Hpo phosphorylation at T195, thereby reducing the Hippo signal transduction cascade (Chen et al., 2014). Moreover, in the fly midbody, CYLD also associates with HDAC6 to regulate the rate of cytokinesis (Massoumi et al., 2006; Wickström et al., 2010). The mutant *Cyld*Δ932 destabilises p53 and consequently some cell cycle genes downstream of p53, as demonstrated in keratinocytes derived from mice expressing this variant (Fernández-Majada et al., 2016).

Therefore, CYLD contributes to control of the cell cycle at different stages: during G1/S transition phase, mitosis and cytokinesis. Important cellular checkpoints that can lead to uncontrolled proliferation and tumorigenesis. Taken together, studying the DUB and non-DUB functions of CYLD has identified mechanisms that support its role as a tumour suppressor, as well as its roles in regulating diverse cellular processes. Consequently, CYLD dysregulation may have multilevel cellular impacts in disease pathogenesis where CYLD is mutated or its expression is lost.

## CYLD in human disease

Given the known widespread roles of CYLD, it is perhaps unsurprising that it has been shown to play a role in a range of human diseases. Loss- and gain-of-function germline pathogenic variants cause skin tumour disorders and neurodegenerative disease, respectively, as outlined below. Work in murine homozygous *Cyld* transgenic models (Box 2, Table 2) has also shown that wide-ranging immune cell functions depend on CYLD, including B- and T-cell homeostasis (Hövelmeyer et al., 2007; Reiley et al., 2006), dendritic cell regulation (Srokowski et al., 2009) and regulatory T-cell function (Lee et al., 2021). Although notably, CCS patients who carry heterozygous pathogenic *CYLD* variants do not appear to present with an immune phenotype (Rajan et al., 2009). However, a recent study has shown *sCYLD* overexpression in gut tissue T-cells from a limited series of patients with inflammatory bowel disease (Tang et al., 2019), indicating that the link between CYLD status and immune function originally described in mice may also exist in patients. In the future, further human diseases may be linked to CYLD, as clinical DNA sequencing and transcriptomics are increasingly used in clinical care (Table 2).

**Table 2.**
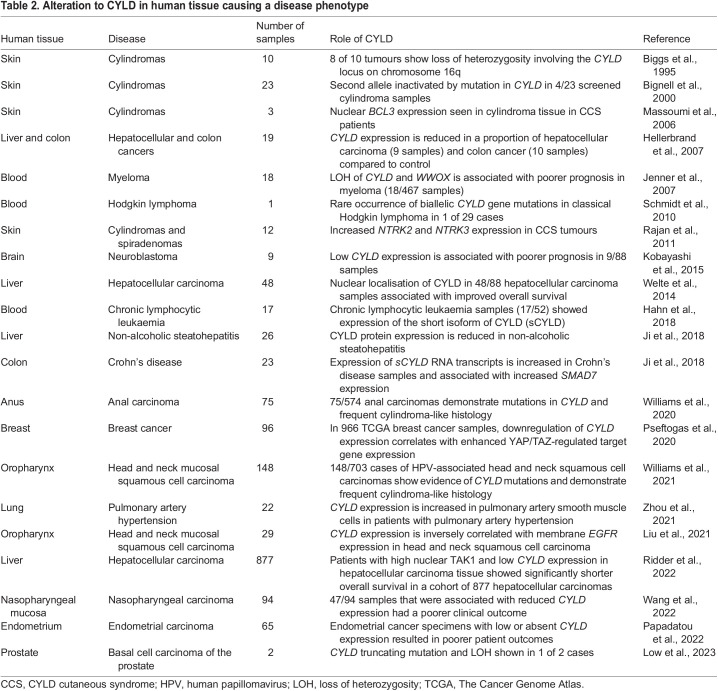
Alteration to CYLD in human tissue causing a disease phenotype

### CYLD-associated germline human diseases

**Fig. 5.**
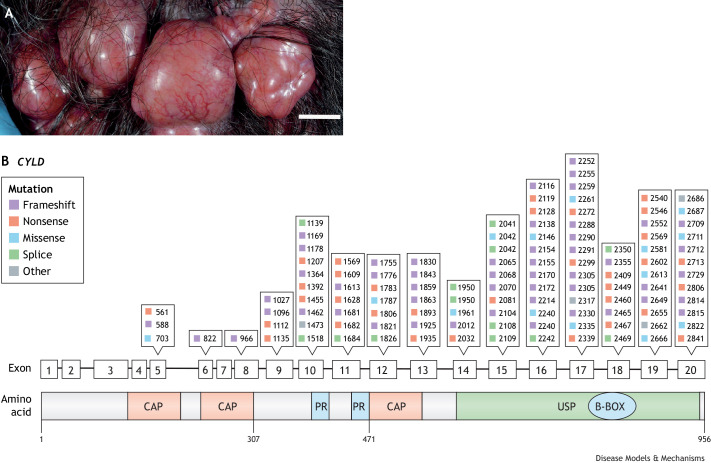
**CYLD cutaneous syndrome (CSS) skin tumours and position of patient mutations in CCS.** (A) A confluent mass of cylindromas affecting the hair-bearing scalp of a CCS patient (scale bar: 1 cm). (B) Germline pathogenic variants reported in CCS. Exons of *CYLD* are shown (middle), with markers denoting reported mutations (top). Corresponding to the exonic structure, the CYLD protein structure schematic (bottom) shows orange boxes indicating the positions of the three CAP-Gly domains and a green box indicating the CYLD catalytic USP domain. Most germline mutations cluster in this domain.

As discussed at the beginning of this article, *CYLD* was discovered to be the gene responsible for CCS, an inclusive label for the inherited skin adnexal tumour syndromes Brooke-Spiegler syndrome (OMIM 605041), familial cylindromatosis (OMIM 132700) and multiple familial trichoepitheliomas (OMIM 601606) (Biggs et al., 1995; Bignell et al., 2000), by studying patient tumour and germline DNA of affected families. All three syndromes arise due to heterozygous germline loss-of-function pathogenic variants in *CYLD*, and the difference in phenotypes seen even within the same family has been ascribed to yet undiscovered modifier genes (Rajan et al., 2009). The overall prevalence of CCS is still unknown but may be in the order of 1 in 100,000 individuals (Rajan and Ashworth, 2015). CCS patients develop multiple benign hair follicle tumours on the head and torso (Rajan et al., 2009), which grow from puberty and accumulate throughout adulthood. The most frequent tumours seen in CCS are cylindromas, spiradenomas and trichoepitheliomas (Fig. 5A). Despite an autosomal-dominant pattern of inheritance, female CCS patients may be more severely affected, and although this has been reported in several families, the underlying mechanism for this disparity is not fully understood (Rajan et al., 2009).

Almost all CCS skin tumours exhibit loss of heterozygosity affecting chromosome 16q12-13, the *CYLD* locus (Blake and Toro, 2009; Davies et al., 2019; Leonard et al., 2001). Almost 90% of the tumour-predisposing germline mutations in *CYLD* are truncating (Blake and Toro, 2009), which affect mostly the USP C-terminal catalytic domain (Forbes et al., 2016; Walker et al., 2015). The most common truncating mutations in CCS are frameshift mutations (39% of overall mutations), which are responsible for approximately half of the reported disease-causing *CYLD* variants (Nagy et al., 2021), followed by nonsense mutations (22%) (Fig. 5B). Missense mutations, which reduce DUB activity, affect almost 10% of affected families. A small proportion of reported pathogenic variants is due to large deletions and rearrangements. Somatic whole-genome sequencing of CCS skin tumours confirms loss of *CYLD* heterozygosity and demonstrates additional mutations in the tumour suppressors *DNMT3A* and *BCOR* in up to a third of benign CCS tumours, and in *TP53* and *MBD4* in some malignant CCS tumours (Davies et al., 2019). *TP53* mutation is a recurrent feature in malignant CCS tumours, suggesting that p53 plays a key role in malignant transformation in CCS (Kazakov et al., 2010).

### Somatic *CYLD* mutation and loss of CYLD expression in sporadic cancers

Beyond CCS, loss of CYLD function is increasingly recognised to play a role in a range of human cancers. Somatic mutations affecting *CYLD* expression beyond the skin were first shown in myeloma, where deletions involving the *CYLD* locus were associated with poorer clinical outcome (Jenner et al., 2007). *CYLD* expression is downregulated through a range of mechanisms in melanoma (Massoumi et al., 2009), breast cancer (Hayashi et al., 2014), haematologic malignancies (Espinosa et al., 2010; Walker et al., 2015), colon cancer, hepatocellular cancer (Hellerbrand et al., 2007), neuroblastoma (Kobayashi et al., 2015) and basal cell prostate cancer (Low et al., 2023) (Table 2). Alternative splicing, resulting in the expression of *sCYLD*, was first discovered in mice (Hövelmeyer et al., 2007). Aberrant expression of sCYLD was subsequently detected in a third of human chronic lymphocytic leukaemias in one study, in which sCYLD was proposed to contribute to chronic lymphocytic leukaemia pathogenesis (Hahn et al., 2018). More recently, recurrent mutations in *CYLD*, predominantly whole-gene deletions and nonsense or frameshift mutations, have been identified in head and neck squamous cell carcinoma (21% of cases) (Williams et al., 2021), thymic carcinoma (10% of cases) (Giaccone et al., 2018) and anal carcinoma (13% of cases) (Williams et al., 2020). In head and neck, as well as in anal carcinoma, *CYLD* mutations were associated with high-risk HPV status and frequent cylindroma-like histopathology. In thymic carcinoma, pathogenic *CYLD* variants were associated with increased PD-L1 (CD274) levels and better response to immune checkpoint inhibitor therapy (He et al., 2021).

Murine models (Box 2) have been used to investigate the cellular mechanisms behind CYLD-driven skin tumour development. For example, primary cells from transgenic mice lacking *Cyld* partially recapitulate the cellular hyperproliferation seen in CCS; and *Cyld*^−/−^ mice treated with 12-O-tetradecanoylphorbol-13 acetate or ultraviolet light are more susceptible to mutagen-induced skin tumours (Jin et al., 2016; Massoumi et al., 2009). In *Cyld*-deficient keratinocytes, excessive ubiquitination of the proto-oncogene *Bcl3* leads to its accumulation in the nucleus, activating NF-κB and inducing transcription of its target genes, such as that encoding cyclin D1 (Massoumi et al., 2006). Transgenic mice overexpressing wild-type *Cyld* under a keratin promoter (K5-*Cyld-*wt) showed reduced activation of the NF-κB pathway in the skin in response to TNFα. When subjected to cellular stress, K5-*Cyld-*wt keratinocytes were more prone to apoptosis, supporting CYLD's role as a tumour suppressor (Alameda et al., 2021). The importance of *Cyld* loss promoting a mitogenic state is underscored in a murine model of melanoma overexpressing *Snail1*, a transcription factor that triggers epithelial–mesenchymal transition, which is associated with tumour progression and invasion (Massoumi et al., 2009). SNAIL1 represses *Cyld* expression, leading to nuclear translocation of BCL-3 and activation of *Ccnd1* and *Cdh2* promoters, driving the proliferation and invasion of melanoma cells in murine models (Massoumi et al., 2009). In a Tg(*Grm1*) mouse model for spontaneous melanoma (Pollock et al., 2003), *Cyld* deficiency promotes cancer onset and growth. Differential gene expression analyses in Tg(*Grm1*)*Cyld*^−/−^ mice revealed alterations in genes involved in cell proliferation, migration and angiogenesis (de Jel et al., 2019). Melanoma cells of Tg(*Grm1*)*Cyld*^−/−^ mice also demonstrate changes in chromatin structure. The loss of *Cyld* led to increased expression of the histone methyltransferase, EHMT2, associated with an increase in the histone methylation mark H3K9me2 and heterochromatin compaction (Schott et al., 2022). A comparison of cells from wild-type and *Cyld*^−/−^ mice confirmed a role for CYLD in chromatin structure dynamics, particularly in euchromatin maintenance. Pharmacological inhibition of EHMT2 rescued the excess heterochromatin compaction observed in *Cyld*^−/−^ melanoma cells (Schott et al., 2022). The mechanistic dissection of the role of CYLD in cancers apart from melanoma is likely to continue to expand and may reveal disease-specific pathogenic roles.

### Neurodegenerative diseases

In 2020, Dobson-Stone et al. identified a heterozygous missense pathogenic variant in *CYLD*, M719V, as the cause of autosomal-dominant FTD and amyotrophic lateral sclerosis (ALS) in a large multigenerational Australian family of European descent (Dobson-Stone et al., 2020). Unlike loss-of-function missense pathogenic variants linked to CCS, CYLD_M719V_ showed a significant increase in K63 deubiquitinase activity and increased inhibition of NF-κB. Primary mouse neurons overexpressing CYLD_M719V_ showed shorter axons and recapitulated cytoplasmic mislocalisation of TDP-43 (TARDBP), a common neuropathological phenotype of FTD and ALS (Dobson-Stone et al., 2020). FTD is a heterogeneous group of disorders that can present clinically with personality and behavioural changes and/or language deficits. There is significant clinical and neuropathological overlap between FTD and ALS, a motor neuron disease leading to progressive weakness and muscle atrophy (Burrell et al., 2016). Some of this overlap is due to shared genetics: pathogenic variants in several different genes can cause FTD and/or ALS, with a repeat expansion in *C9orf72* being the commonest genetic cause of both disorders (DeJesus-Hernandez et al., 2011; Renton et al., 2011). Other rarer causes of FTD and/or ALS include pathogenic variants in the genes encoding p62, TBK1 and optineurin, which are direct interactors with CYLD (Cirulli et al., 2015; Fecto et al., 2011; Freischmidt et al., 2015; Maruyama et al., 2010). CYLD_M719V_-linked FTD and ALS may therefore be mediated via altered interactions with or processing of one or more of these proteins (Friedman et al., 2008; Jin et al., 2008; Nagabhushana et al., 2011). More broadly, autophagy and cytoskeletal dynamics have been identified as dysregulated in ALS (Weishaupt et al., 2016), and thus CYLD involvement in these cellular processes as outlined above may be relevant.

In addition to M719V, several rare *CYLD* missense variants have been identified in other European and Chinese ancestry patients with clinically diagnosed FTD (Dobson-Stone et al., 2020; Tábuas-Pereira et al., 2020; Xiao et al., 2022), ALS (Dobson-Stone et al., 2020; Gu et al., 2021) and Alzheimer's disease (Oyston et al., 2020; Xiao et al., 2022). In the absence of DNA samples from affected family members to demonstrate segregation or of *in vitro* functional analyses, however, it is premature to draw conclusions about the neuropathogenicity of these rare missense variants.

These genetic correlations have prompted researchers to study the effects of aberrant CYLD on neurophenotypes in murine models. The phenotypic consequences of mutations or tissue-specific alterations in *Cyld* expression from several animal models demonstrate close links between CYLD and neurodegeneration. Mouse models in which *Cyld* is either overexpressed or knocked out show that CYLD signalling via AKT–mTOR-mediated autophagy acts as a synaptic autophagy activator to modulate synapse maintenance, function and plasticity (Zajicek et al., 2022). A separate study showed that loss of *Cyld* results in major autism-like phenotypes in mice, including impaired social communication, increased repetitive behaviour and cognitive dysfunction. These behavioural phenotypes are correlated with reductions in hippocampal network excitability, long-term potentiation and pyramidal neuron spine numbers (Colombo et al., 2021). Additionally, *Cyld* knockout impairs amygdala-dependent tone-cued fear memory, as *Cyld*^−/−^ mice exhibit disrupted neuronal activity and synaptic transmission in the basolateral amygdala and a concomitant impaired fear memory (Li et al., 2021b). *Cyld* knockout mice also have a mild hearing impairment, or auditory neuropathy, perhaps associated with reduced neurite outgrowth (Yang et al., 2021).

Interestingly, loss of *CYLD* can also be neuroprotective. *Cyld* knockout mice showed less secondary neurodegeneration after traumatic brain injury than did wild-type mice with the same injury (Ganjam et al., 2018). And neuronal-specific depletion of *dCYLD* in *Drosophila* accelerates Parkin-interacting substrate (Paris) ubiquitination-dependent degradation. Excess Paris accumulation leads to mitochondrial biogenesis defects in fly dopaminergic neurons, and reduced dCYLD levels therefore exert a neuroprotective effect against Parkinson's disease-like neurodegeneration (Pirooznia et al., 2022). These mitochondrial effects are proposed to couple CYLD activity to dopaminergic neurodegeneration and behavioural deficits observable in *Prkn* knockout mouse models (Pirooznia et al., 2022).

## Future directions for human translation

Despite the advances in CYLD modelling, unanswered questions pose a challenge for the field. It remains unclear as to why the expression of CCS patient-derived pathogenic variants of *Cyld* in murine skin does not give rise to cylindromas (Jin et al., 2016). This may reflect differences in hair follicle biology between mice and humans, additional hormonal factors for CCS tumour induction in humans, or perhaps the requirement for *CYLD* deficiency in a specific stem cell in hair follicles that is key for tumour formation. Nonetheless, the lack of a model that recapitulates the human CCS tumour phenotype hampers drug discovery and translational advance. Patient-derived xenograft modelling may be a route (Andersson et al., 2019), but poses challenges given the rarity of fresh CCS tumour material. Beyond skin, the *CYLD* variant-related neurodegenerative phenotype seen in humans has prompted a new direction of investigation, with animal modelling already suggesting an important role for CYLD in unexpected phenotypes such as autism-like behaviours. The role of CYLD in other diseases such as Parkinson's disease, in which it has been shown to impact mitochondrial proteins in dopaminergic neurons, has brought interesting links to mitochondrial biogenesis. Ubiquitin-editing enzymes therefore continue to be an exciting area for discovery, and CYLD promises to be an interesting enzyme to watch.
